# Computational identification of obligatorily autocatalytic replicators embedded in metabolic networks

**DOI:** 10.1186/gb-2008-9-3-r51

**Published:** 2008-03-10

**Authors:** Ádám Kun, Balázs Papp, Eörs Szathmáry

**Affiliations:** 1 Collegium Budapest, Institute for Advanced Study, Szentháromság utca 2, Budapest H-1014, Hungary; 2 Department of Plant Taxonomy and Ecology, Institute of Biology, Eötvös University, Pázmány Péter sétány 1/C, Budapest H-1117, Hungary; 3 Faculty of Life Sciences, The University of Manchester, Oxford Road, Manchester M13 9PT, UK; 4 Parmenides Center for the Study of Thinking, Kardinal Faulhaber Strasse, Munich D-80333, Germany; 5 Current address: Institute of Biochemistry, Biological Research Center, Szeged H-6701, Hungary

## Abstract

**Background:**

If chemical A is necessary for the synthesis of more chemical A, then A has the power of replication (such systems are known as autocatalytic systems). We provide the first systems-level analysis searching for small-molecular autocatalytic components in the metabolisms of diverse organisms, including an inferred minimal metabolism.

**Results:**

We find that intermediary metabolism is invariably autocatalytic for ATP. Furthermore, we provide evidence for the existence of additional, organism-specific autocatalytic metabolites in the forms of coenzymes (NAD^+^, coenzyme A, tetrahydrofolate, quinones) and sugars. Although the enzymatic reactions of a number of autocatalytic cycles are present in most of the studied organisms, they display obligatorily autocatalytic behavior in a few networks only, hence demonstrating the need for a systems-level approach to identify metabolic replicators embedded in large networks.

**Conclusion:**

Metabolic replicators are apparently common and potentially both universal and ancestral: without their presence, kick-starting metabolic networks is impossible, even if all enzymes and genes are present in the same cell. Identification of metabolic replicators is also important for attempts to create synthetic cells, as some of these autocatalytic molecules will presumably be needed to be added to the system as, by definition, the system cannot synthesize them without their initial presence.

## Abstract 2

Small-molecular metabolic autocatalytic regulators, which are crucial to metabolic pathways, are identified in a novel systems-wide study in different organisms, revealing that in the enzymatic reactions of conserved autocatalytic cycles, the autocatalytic behavior of replicators varies.

## Background

Two fundamental features of living systems are heredity and metabolism, the latter being controlled by the former [1-3]. Although heredity is often considered to be exclusively dependent on template replication of nucleic acid polymers, it is not the only way of storing and transmitting information. Membranes [4] and epigenetic chromatin-markings [5], for example, are considered to be replicators providing a limited but important part of cellular inheritance. From the chemical point of view the essence of replication is autocatalysis [6], that is, when a compound catalyses its own formation (for example, DNA is needed for the synthesis of more DNA). One key model of minimal life [7] suggests that in addition to template replication and membrane growth, metabolism is also autocatalytic and, hence, results in replication.

In a trivial sense the cytoplasm is autocatalytic in that just the membrane and DNA alone are incapable of replication. DNA may code for the constituents of cytoplasm, but without these constituents there is no machinery to do anything. Enzymes would need to be added to the system. Enzymes catalyze the synthesis of more enzymes, so the enzymatic machinery can be regarded as autocatalytic. However, according to Gánti's theory [1,8,9], metabolism is autocatalytic at the level of small molecules (intermediates) as well, hence mere addition of enzymes and raw materials should be unable to kick-start the system. This being so we should be able to identify autocatalytic components of metabolism that are, in effect, replicators. It has been pointed out that the Calvin cycle [1] and the reductive citric acid cycle [10] are such autocatalytic networks.

As Gánti [7] pointed out, any member of an autocatalytic cycle is an autocatalyst. Thus, the reductive citric acid cycle can be launched with any of the intermediates, including, for example, fumarate, succinate, citrate, or oxaloacetate. The same is true of metabolic networks, but networks present additional complications because of their complicated stoichiometric structure. Consider the Calvin cycle as analyzed by Gánti [11]. If we provide the system with the necessary coenzymes (including ATP) and CO_2_, one molecule of 3-phospho-glycerate is still not sufficient for autocatalytic growth: one needs three molecules of 3-phospho-glycerate to produce a fourth one, and, ultimately, three new molecules in addition to the three with which the system was successfully launched. But since we are dealing with a network, alternative starting compound sets are possible, such as (xylulose-5-P AND erythrose-4-P), OR (dioxyacetone-P AND 3-P-glyceraldehyde AND erythrose-4-P). The more complex the autocatalytic network, the more alternative sets we can expect to be identified, but this expectation is reduced by the also increasing number of interconversions due to alternative reaction pathways in large systems. But in general a few alternative obligatorily autocatalytic sets can be expected to be present.

**Figure 1:**
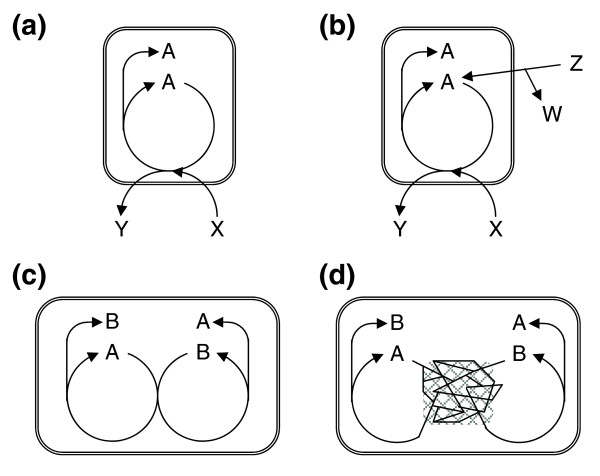
Various metabolic organizations. **(a) **A protocell showing an indispensable autocatalytic metabolite. **(b) **A richer medium is able to kick-start metabolism because A can be formed from Z. **(c) **The set of autocatalytic molecules is composed of A and B, a pair of cross-catalytic molecules (that is, A is required to kick-start the biosynthetic route to B, and B is required to kick-start the biosynthetic route to A). Although the excess molecules are produced in a mirror fashion, it is easy to identify the autocatalytic compounds. **(d) **If reactions involving A and B are embedded in a large network, it may not be easy to identify the autocatalytic compounds: note that the excess A is produced remote from where it was consumed in the first place. Symmetrically, the same holds for B. Inner metabolite A, food X and Z, waste Y and W.

The relevance of these cycles is not clear, however, as these are embedded in larger networks of reactions, through which the cycle intermediates could potentially be reconstructed. Thus, the existence of an autocatalytic sub-network does not guarantee that the whole network is autocatalytic (Figure 1). Conversely, lack of an obvious smallish (easy-to-identify) autocatalytic cycle does not prevent the whole metabolism from being autocatalytic as several auto- and cross-catalytic (Figure 1c) molecules might be present in the network, which can be produced remote from where they are consumed in the first place (Figure 1d). We can reformulate the empirical question as follows: are the intermediates of a given metabolic network accessible just from the raw materials (members of a food set), or does one need to add some molecule(s) from the network itself? It is obvious that the answer will depend on the nature of the organism and the specified food set. A richer medium may allow the synthesis of compounds that would otherwise be inaccessible without some help 'from within' (that is, an autocatalytic component; Figure 1). From now on we call a metabolic network autocatalytic if at least one additional non-food metabolite must be added to the network of an organism to render it complete so that all its known reactants become accessible by a series of biochemical reactions. Importantly, a mere kinetic autocatalytic effect of, say, a cofactor, is not sufficient to include it in the set of autocatalytic compounds: if this compound can be synthesized by at least one alternative pathway just from the food set and other autocatalytic molecules, then we do not add it to the set of autocatalytic compounds. Thus, the autocatalytic metabolite set of an organism includes all compounds that can be synthesized by small molecule metabolism, have an autocatalytic nature, and that must be present within the cell because otherwise the network, or part of it, would halt. We refer to such cases as obligate autocatalysis.

Another intellectual source behind the present study is an earlier nutrient-related analysis of *Escherichia coli *metabolism by Romero and Karp [12] aiming at identifying incomplete regions of a pathway database. Although the authors identified what they called 'bootstrapping molecules' (that is, those required to bootstrap the entire metabolism), these are typically not the same as those in our autocatalytic metabolite sets. For example, vitamin B12 cannot be synthesized by *E. coli*, thus it is a bootstrapping molecule *sensu *Romero and Karp, but is clearly not an autocatalytic metabolite by our definition. Moreover, the authors identified the set of bootstrap molecules with the preconception that certain compounds should necessarily be bootstrapping. Thus, it remains to be investigated in an unbiased way whether large metabolic networks contain autocatalytic components.

In the present study we test the idea that intermediary metabolisms of extant organisms are autocatalytic and we attempt to systematically identify autocatalytic sub-networks in different species using recently published high-quality metabolic network reconstructions. Our analysis not only shows that ATP is a universal obligatory autocatalytic compound, but also reveals species-specific differences in the set of autocatalytic molecules and variations in the structure of some autocatalytic sub-networks. These findings lend strong support to the view that replication in living systems is not restricted to macromolecules, but also involves small-molecule metabolism [1,8,9], albeit with limited heredity [13].

## Results

### Identification of obligate autocatalytic metabolites

We investigated numerous metabolic networks capable of uptaking different sets of nutrients, including the network of an autotrophic species (a cyanobacterium) and also a hypothetical minimal network (Table 1). Since accurate information on cofactor usage and transport processes might be crucial to correctly identify autocatalytic compounds, we included only high-quality, manually reconstructed genome-scale metabolic networks (except for *Synechocystis *sp. PCC 6803 and the hypothetical minimal metabolism where such reconstructions were not available). In contrast to high-throughput automated reconstructions, these published networks were manually reconstructed from diverse information sources and contain accurate information on reaction reversibility, cofactor usage and also include transport processes and reactions without assigned open reading frames [14]. In the case of *Synechocystis *sp. PCC 6803 we attempted to reconstruct a genome-scale metabolic network from the publicly available automatic reconstruction of MetaCyc [15] as a template. Reaction reversibility and cofactor usage were determined by comparison with manually curated networks. The reaction network was further refined based on other databases and data from the literature (Additional data file 1). The hypothetical minimal metabolic network investigated here is based on the one proposed by Moya and co-workers [16].

**Table 1:** List of investigated metabolic networks and their main properties

	Total number of metabolites	Number of producible metabolites (maximum scope)	Number of food molecules	Scope of input metabolites	Additional metabolites to include for maximum scope*	Reference
*Escherichia coli*	761	692	143	315	ATP	[25]
*Heliobacter pylori*	485	441	74	182	ATP	[45]
*Staphylococcus aureus*	644	543	83	194	ATP	[46]
*Saccharomyces cerevisiae*	672	667	101	342	ATP	[47]
*Lactococcus lactis*	508	477	92	190	ATP	[48]
*Streptomyces coelicolor*	601	562	104	267	ATP	[49]
*Mycobacterium tuberculosis*	830	642	87	235	ATP	[50]
*Methanosarcina barkeri*	628	566	70	161	ATP + NAD^+^	[51]
*Geobacter sulfurreducens*	541	406	41	82	ATP + NAD^+ ^+ THF + CoA	[52]
*Synechocystis*^†^	879	634	18	64	ATP + NAD^+ ^+ THF + CoA + sugar	^‡^
*Synechocystis*^§^	879	662	29	99	ATP + NAD^+ ^+ THF + CoA	^‡^
Minimal metabolism	68	68	11	11	ATP	[16]

*For a list of equivalent molecules that give the same scope see Additional data file 1. ^†^Autotrophic growth. ^‡^Heterotrophic growth. ^§^See Additional data file 1 and Additional data file 3.

We performed computational analyses of the metabolic networks to identify autocatalytic compounds, that is, intermediate metabolites that are required for their own biosynthesis and, therefore, cannot be accessed from the food set. Given an initial set of metabolites (seed set), the method of scope analysis [17,18] allows us to find all the metabolites that can be accessed from these initial molecules given a list of biochemical reactions (note that the method has been successfully applied to the problem of the impact of oxygen on the evolutionary extension of metabolism [19]). A metabolite is considered accessible if all the substrates of at least one of the reactions producing this metabolite are present. The initial set consisted of all possible compounds that can be imported from the external environment via transport reactions (food set) and those macromolecules that participate in some reactions but cannot be synthesized by the network (for example, acyl-carrier protein, ferredoxin, and so on; see Additional data file 1 for a list for each species). In the case of *Synechocystis*, we also defined a food set comprising only inorganic compounds required for autotrophic growth. Generally, an analysis starting from the richest medium (as defined by the full complement of transportable nutrients) would identify the minimum set of autocatalytic metabolites, whereas an investigation starting from a minimal medium would identify the largest set of autocatalytic molecules for a given organism. As biosynthetic pathways leading to certain compounds are still not completely characterized in the available metabolic reconstructions, we cannot expect the scope of the initial molecules to span all metabolites, even if otherwise no autocatalytic metabolite is present in the network. To circumvent this difficulty, we identified the sets of molecules that can be produced by each metabolic network (see Materials and methods). If the scope of the external molecules did not extend to all producible molecules, then we searched for the internal molecule whose addition to the initial seed increased the scope the most. Next, this molecule was added to the initial seed and the scope analysis was repeated. We continued to add molecules until the scope of the seed matched the set of producible compounds. Finally, we inferred the smallest set of autocatalytic compounds for each network based on the results of the scope analysis (see Materials and methods; Additional data file 1).

### ATP is an obligate autocatalyst in metabolism

Our systematic analysis reveals that in none of the 11 investigated metabolic networks did the scope of the externally available molecules include all producible compounds (Table 1), therefore providing evidence that the metabolic networks of these organisms are autocatalytic. At least one small molecule has to be invariably provided for the metabolism to be kick-started: we found that the presence of internal ATP (or an equivalent compound; Additional data file 1) is required in all studied networks. Moreover, in eight networks addition of ATP alone to the initial seed was sufficient to reach all producible intermediate metabolites. The autocatalytic nature of ATP synthesis in isolation is apparent in glycolysis [20] and was used in industrial biochemistry 40 years ago [21]. Other autocatalytic routes of ATP synthesis have also been described [22]. Furthermore, our results for yeast (*Saccharomyces cerevisiae*) suggest that eukaryotic cells bear at least two autocatalytic compounds: cytoplasmic and mitochondrial ATP. This finding is entirely consistent with the endosymbiotic origin of mitochondria and demonstrates that the mitochondrion retained not only its genetic membranes [4], but also its metabolic replicator for hundreds of millions of years. Our finding that the mitochondrial ATP pool is autocatalytic despite the presence of an ATP-ADP translocator in the mitochondrial membrane suggests that ATP would qualify as a metabolic replicator even in those intracellular parasites capable of ATP uptake via ATP-ADP exchange (for example, *Chlamydia psittaci *[23]). Although the lack of metabolic reconstructions for such parasitic organisms hindered us from directly testing this possibility, we could still investigate the idea by including a fictive ATP-ADP exchange reaction in the *E. coli *network (ADP + ATP[external] + Pi + H ↔ H[ext] + ADP[ext] + ATP + Pi[ext]) and adding external (ext) ATP and ADP to the food set. Notwithstanding these modifications, we still identified ATP as an autocatalytic molecule, which can be explained by the fact that ADP/ATP must be simultaneously present on both sides of the membrane for the transport reaction to run. In summary, our results show that, to our present knowledge of metabolisms, the autocatalytic synthesis of ATP is unlikely to be bypassed by other reactions in a larger network.

### Organism-specific autocatalytic compound sets

**Figure 2:**
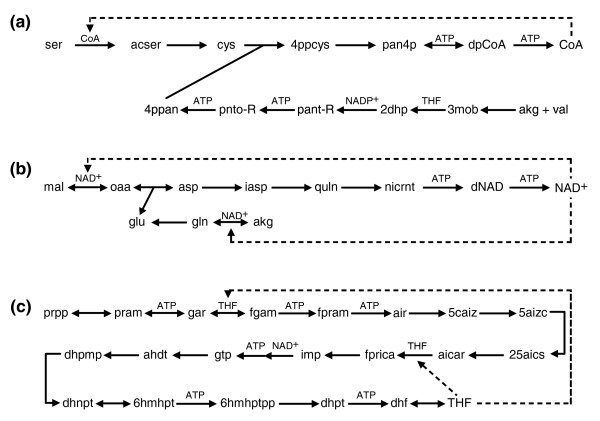
Autocatalytic synthesis of coenzymes in *Synechocystis *sp. **(a) **Coenzyme A. **(b) **NAD^+^. **(c) **Tetrahydrofolate. Autocatalytic metabolites involved in a reaction are indicated above the arrows. Dashed lines point to the reactions where the autocatalytic metabolites are involved in their own synthesis. See Additional data file 3 for the full names of metabolites.

In three of the investigated networks ATP is not the only necessary autocatalytic compound. As expected, the largest number of autocatalytic metabolites is present in the photoautotrophic species *Synechocystis *sp., which requires only a limited set of inorganic food molecules for autotrophic growth. Analysis of the metabolic network of *Synechocystis *sp. strain PCC6803 reveals four additional autocatalytic sub-networks (Figure 2). The Calvin cycle is clearly autocatalytic when the food set comprises only inorganic compounds: sugar is needed to fix CO_2 _and produce more sugars. As different sugars are inter-convertible, any one of 138 different molecular species can fulfill this requirement. The Calvin cycle, however, does not remain autocatalytic upon inclusion of organic compounds in the food set (Additional data file 1). Furthermore, the biosynthesis of NAD^+^, coenzyme A (CoA) and tetrahydrofolate (THF) was also found to be autocatalytic in *Synechocystis*, irrespective of the food set (Table 1, Figure 2).

Apparently, the reactions of the autocatalytic cycles identified in *Synechocystis *(with the exception of the Calvin cycle) are present in most of the studied organisms (Table 2): for instance, enzymes of CoA biosynthesis are found in all studied species. However, these metabolic routes do not necessarily operate as autocatalytic sub-networks in other organisms, either due to the possibility to uptake certain intermediates from the environment or due to the presence of enzymatic reactions leading to key intermediates. To further investigate this issue, we repeated the analysis of the *E. coli *network under a condition where only glucose and inorganic compounds were included in the food set (that is, a minimal medium). We found that in addition to ATP, NAD^+^, CoA and quinones also behave as autocatalytic compounds under this condition (Additional data file 1), demonstrating that uptake of certain intermediates from the environment can kick-start the corresponding autocatalytic sub-networks. For example, uptake of cysteine renders biosynthesis of CoA non-autocatalytic in *E. coli*. On the other hand, the fact that we did not identify THF as an autocatalytic metabolite in this species can best be explained by structural differences between the *Synechocystis *and *E. coli *networks: the possibility to convert AMP to GMP via IMP in the nucleotide salvage pathway of *E. coli *renders THF synthesis non-autocatalytic, even when folate is absent from the medium.

**Table 2:** The presence/absence of pathways involved in the biosyntheses of potentially autocatalytic cofactors

	NAD^+^	CoA	THF
*Escherichia coli*	+	+	+
*Heliobacter pylori*	+*	+	+
*Staphylococcus aureus*	P	+	+
*Saccharomyces cerevisiae*	P	+	+
*Lactococcus lactis*	P	+	+
*Streptomyces coelicolor*	+	+	+
*Mycobacterium tuberculosis*	+	+	+
*Methanosarcina barkeri*	P	+	P
*Geobacter sulfurreducens*	+*	+	+
*Synechocystis*	+	+	+
Minimal metabolism	P	P	P

A plus sign (+) indicates the biosynthetic route is fully present in the network; 'P' indicates the biosynthetic route is partially present in the network; asterisks indicate the biosynthetic route is slightly different from the one found in *Synecocystis *sp.

### Alternative forms of metabolic replicators

Some autocatalytic compounds of *Synechocystis*, however, remain autocatalytic in certain heterotrophic organisms, despite the fact that all transportable nutrients are included in the food sets (see Table 1 for examples). Nevertheless, even if biosynthesis of the same molecule proves to be autocatalytic in two different organisms, species-specific differences in the organization of the autocatalytic sub-networks can be observed in some cases. For instance, NAD^+ ^is an autocatalytic metabolite in both *Methanosarcina barkeri *and *Geobacter sulfurreducens*, but NAD^+ ^(or NADH) is required for its own synthesis in different biochemical reactions in the two organisms (Figures S3 and S6 in Additional data file 1), hence providing evidence for the existence of alternative forms of metabolic replicators, where the whole relevant cycle or network constitutes the autocatalyst.

### Equivalent compounds in autocatalytic coenzyme synthesis cycles

**Figure 3:**
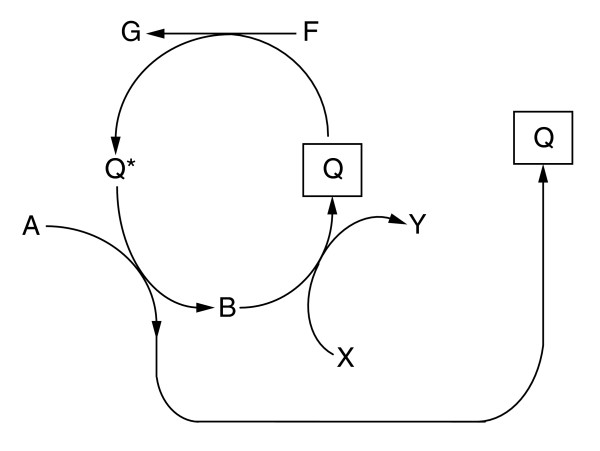
General scheme for the autocatalytic synthesis of a coenzyme. The coenzyme (carrier molecule) and its loaded form are denoted by Q and Q*, respectively. A, X and F are external compounds provided to the cycle. B is an intermediate in the cycle. G and Y are by-products of the cycle. B can be considered an autocatalyst if X is provided as an external compound. However, if X is an intermediate of the whole network (that is, an internal compound) then providing B does not necessarily launch the cycle because biosynthesis of X might require the presence of coenzyme Q*.

In line with the general considerations on autocatalytic cycles, we find that ADP and ATP are both autocatalysts since they are intermediates of the same autocatalytic cycle [11]. However, analysis of autocatalytic coenzyme synthesis in general is a challenge. Following the notation of Gánti [7], let the loaded form of a coenzyme be Q*, and the carrier molecule be Q (for example, the NADH:NAD^+^, acetyl-CoA:CoA, ATP:ADP pairs). Consider the following imaginary biosynthesis of this coenzyme (Figure 3). Suppose external compounds A, X and F are provided to this cycle. This looks like an ordinary autocatalytic cycle with asymmetric branches leading to the copies of Q (similar to the topology of the reductive citric acid cycle). If so, B is also an autocatalyst. If B could be synthesized from other external materials, Q and B would cease being obligatory autocatalytic. Let, however, X be an intermediate of the whole network, thus an internal compound. In this case providing B does not settle the issue, because now we should look into the synthesis of X in other parts of the whole network. Consider, for example, the topology of the autocatalytic synthesis of NAD^+ ^as shown in Figure S1 in Additional data file 1. It suggests that aspartate could replace NAD^+^, and seemingly the same applies to glutamate. However, launching the NAD^+ ^synthesis cycle with glutamate also requires oxaloacetate, and the latter requires NAD^+^, hence glutamate cannot replace NAD^+ ^in the obligatorily autocatalytic set, but aspartate can. Thus, identifying the sets of equivalent autocatalytic compounds embedded in large networks requires a systems-level approach. We provide a list of equivalent autocatalytic metabolite sets (that is, sets of autocatalytic compounds, usually forming reaction cycles, where the initial presence of any one compound is sufficient to render the others accessible) for different compound families and different organisms in Additional data file 1.

### Insensitivity of the results to network completeness and accuracy

While we have investigated only high-quality metabolic reconstructions, the completeness of these networks is nevertheless expected to vary between organisms. To assess the sensitivity of our results to different levels of refinement, we repeated our analysis for three increasingly detailed reconstructions of the *E. coli *metabolic network containing 660, 904 and 1,260 genes, respectively [24-26]. Analyses of the three reconstructions gave identical results (data not shown), suggesting that our method is not particularly sensitive to network completeness, at least in *E. coli*. However, in organisms with less-studied metabolism, the set of identified autocatalytic molecules might change if novel metabolic routes and bypasses will be discovered. For example, we found that THF biosynthesis would not be autocatalytic in *G. sulfurreducens *if a route from adenine to IMP were present, as can be inferred from the genome sequence of *Geobacter metallireducens *(based on information in the KEGG database [27]). In a similar vein, the autocatalytic nature of THF should be revisited in *Synechocystis *sp. PCC 6803 once a more complete reconstruction of purine metabolism is available for this species.

In addition to network completeness, we also investigated how the accuracy of reconstruction affects our results. First, biochemical studies show that some ATP utilizing enzymes can also accept GTP (or other NTPs; for example, [28,29]), albeit in a species-specific manner [30,31]. To assess the impact it might have on our results, we analyzed the extreme case when all ATP utilizing reactions of the *E. coli *metabolic network could also use GTP as a cofactor. In this case, GTP (besides ATP) was also identified as an autocatalytic compound; however, this finding does not alter our main conclusion that a nucleotide triphosphate is indispensable to kick-start the metabolism.

Second, we asked how the assignment of reaction reversibility could affect our results. In high-quality metabolic reconstructions, reaction reversibility reflects the direction(s) of the reactions under physiological conditions. On the other hand, any chemical reaction is, in principle, reversible. Thus, one might argue that a very small amount of ATP (or other autocatalytic molecule) could be synthesized by ATP consuming reactions, which could kick-start the metabolism even if ATP was initially absent from the cell. However, we found that two or more 'irreversible' reactions should operate in the reverse direction to produce ATP from food molecules, even in *Mycobacterium tuberculosis *where AMP can be accessed from the food set. Moreover, because at least one of these reactions is always a hydrolysis, and water would be abundant in an 'empty' system, we conclude that, for all practical purposes, these routes can be considered irreversible and production of ATP would be highly unlikely. However, even if a small amount of ATP emerged via slow backward reactions, this would not be sufficient to leave that trivial, non-physiological steady state according to theoretical studies of the dynamics of energy metabolism [20,32]. Thus, although it is out of the scope of our current analysis to analyze the dynamic behavior of autocatalyic subnetworks embedded in large systems, we expect that in addition to network structure, kinetic effects might also contribute to the autocatalytic behavior of certain compounds. Finally, we note that although our computational approach directly identified compounds that are needed to kick-start an 'empty' system (that is, a non-physiological situation), the very same molecules are expected to be synthesized autocatalytically under physiological conditions as well.

## Discussion

We performed systems-level analysis of diverse metabolic networks to demonstrate that intermediary metabolisms contain obligatory autocatalytic biochemical cycles and, hence, qualify as replicators [6]. We found that intermediary metabolism is obligatorily autocatalytic for ATP (even if the system is able to uptake ATP via ATP-ADP exchange). Conceptually, our finding lends support to the view that a small but crucial part of inheritance is provided by the autocatalytic molecules of metabolism [1]. In sharp contrast to DNA-based replication, however, autocatalytic metabolic cycles are not modular replicators since replication is not based on the successive addition of modules, but rather proceeds progressively [13]. Moreover, although nucleic acids have practically unlimited potential to store information (the number of sequence types vastly exceeds the number of individuals in any realistic system), autocatalytic networks of metabolites can have very limited heredity only because the number of alternative types is likely to be small [13]. Our finding, that different forms of autocatalytic sub-networks are associated with NAD^+ ^biosynthesis in two different organisms (Figures S3 and S6 in Additional data file 1) demonstrates that alternative forms of metabolic replicators is not a mere hypothetical possibility [33]. However, the evolutionary role of this variation remains questionable since rival variants should be present in the same population for competition and natural selection to take place. In contemporary systems the metabolic pathways are defined by highly specific catalytic activities provided by the genetically encoded enzymes. It is an open question whether alternative metabolic replicators can exist in their absence - today, the only known such replicator is the formose reaction [34,35] (producing sugars autocatalytically from formaldehyde). However, the formose reaction is non-informational [6]: no alternative cycles are known that would propagate themselves in a hereditary fashion.

The result that even heterotrophs contain metabolic replicators deserves special attention. For autotrophs the presence of clearly autocatalytic sub-networks as the Calvin cycle or the reductive citric acid cycle suggested that at least one of its intermediates, or some related compound, would be in the set of autocatalytic metabolites, given that there are no alternative synthetic routes, which is an empirical issue. We have settled this issue for *Synechocystis *in favor of sugar metabolism being truly autocatalytic in the autotrophic mode. In a similar vein, it will be interesting to examine in the future the autocatalytic compounds of autotrophic microbes running the reverse citric acid cycle (itself also being an autocatalytic cycle for CO_2 _fixation). In contrast, the presence of metabolic replicators in heterotrophs may seem less obvious, since they consume organic compounds in the food set - yet we find at least ATP to be always such a replicator, and occasionally other coenzymes also.

Could the presence of coenzyme replicators be an ancestral feature of intermediary metabolism? As King [36] observed a while ago, the biosynthesis of coenzymes seems to be auto- and cross-catalytic and this may be partly due to ancient metabolic history. This also seems to hold partially in our analysis: for example, THF and NAD^+ ^are needed in CoA synthesis (Figure 2). Nucleotide coenzymes may well be molecular fossils [37] from an RNA world [38]. Considering the fact that they participate in so many reactions and that it would be very hard to replace them after the evolutionary build-up of the enzymatic system, their auto- and cross-catalytic nature indeed speaks for their primitive ancestry in metabolism [33,39]. The fact that comparative analysis of reduced endosymbiont genomes does not suggest coenzyme synthesis in top-down-derived minimal organisms [40] is no argument against such ancestry. If we accept that most of the coenzymatic biochemical reactions cannot be run at an acceptable speed for a primitive cell without the coenzymes, then the only remaining option is a heterotrophic uptake of the precursors of these coenzymes (compare [16]). But unless all the coenzyme precursors (vitamins) were abiogenically synthesized in an environment chemically different from the primitive protocells, the latter may have just been running the needed reactions inside. Early coenzyme synthesis, just as primitive metabolism in general [41], may have been closer to primordial chemistry rather than modern biochemistry. The fact that suggestions for reconstructed minimal cells thriving under nutrient-rich conditions do not contain coenzyme synthesis does not imply that the last universal common ancestor did not have coenzyme synthesis. Indeed, a recent estimate of the gene content of the last universal common ancestor reveals that it might have possessed a fairly complex genome similar to those of free-living prokaryotes, including genes encoding certain enzymatic steps of NAD^+^, CoA and THF biosynthesis [42]. With time, an originally autocatalytic metabolic compound may cease to remain such, as novel routes of synthesis, based on a reduced set of autocatalytic molecules, are discovered by genetic evolution. If this option is not available, the only solution is to evolve an alternative, still autocatalytic, synthetic pathway (analogous to the replacement of one enzyme, taking part in DNA replication, by another).

Our finding that even a minimal metabolism is autocatalytic at the level of small molecules has important implications for attempts to design a synthetic cell. Most efforts to build an artificial self-reproducing system from scratch have focused on constructing simple chemical supersystems capable of template replication and membrane growth, but lacking a metabolic subsystem (see [43] for a review). However, future aims to design a synthetic cell with complex intermediary metabolism should incorporate our findings on the existence of autocatalytic compounds. Moreover, future studies should address the question of whether gene regulatory and signaling networks contain autocatalytic components analogous to those found in metabolism (for example, the product of a positive feedback loop [5]). Thus, an extension of our network-based approach could be used to identify the minimal set of cellular network components that should possibly be provided to kick-start an artificial cell.

## Conclusion

The current study constitutes, to our knowledge, the first systematic search for replicators embedded in large biochemical networks. Although parts of metabolism that are autocatalytic in isolation (for example, Calvin cycle, glycolysis) have been put forward previously, it remained unknown whether these cycles operate in an obligatorily autocatalytic manner when embedded in larger networks. Our analysis of the small molecule metabolism of 10 living organisms and an inferred minimal metabolism suggests that all metabolic networks have at least one universal autocatalytic molecule, ATP (or equivalent compounds). Conceptually, this finding supports the view that a small but important part of inheritance is provided by the set of autocatalytic compounds of intermediary metabolism. Although ATP appears to be the only universal autocatalytic metabolite, other, organism-specific autocatalytic molecules have been identified in the forms of nucleotide cofactors (such as CoA, NAD^+ ^and THF) and sugars or sugar-containing compounds (in the autotrophic metabolism of a photosynthetic bacterium). Importantly, the metabolic pathways associated with these autocatalytic nucleotide cofactors are present in many organisms, but they do not necessarily operate in an autocatalytic manner, as the autocatalytic compounds can be synthesized from food molecules or with the help of alternative pathways. This finding clearly underlines the need for a systems-level approach to identify obligate replicators embedded in large metabolic networks. Our work also has relevance for attempts to create synthetic cells, as some of these autocatalytic molecules will presumably be needed to be added to the system as the system cannot synthesize them without their initial presence.

## Materials and methods

### Identifying the set of producible metabolites

As biosynthetic pathways leading to certain metabolites are still not completely characterized in the available reconstructions, we cannot expect these molecules to be accessible from the food set, even if otherwise no autocatalytic metabolite is present in the network. Thus, before performing the scope analysis, we first need to identify the sets of molecules whose net synthesis is possible in steady state (that is, producible metabolites). Note that those compounds, which cannot be synthesized from the food molecules in steady state would always be identified as inaccessible by the scope analysis (a non-steady-state approach), but the reverse is not necessarily true. Because flux balance analysis is widely used to assess the production capabilities of metabolic networks, we performed a series of flux balance analyses on each network to identify the set of producible metabolites in each organism. As the principles of flux balance analysis have been described elsewhere [44], here we only briefly note that it involves two fundamental steps: first, specification of mass balance constraints around intracellular metabolites (that is, assumption of steady-state); and second, maximization of the production of one or more compounds using linear programming. The assumption of a steady state of metabolite concentrations specifies a series of linear equations of individual reaction fluxes. Availability of nutrients and directions of individual reactions were included as boundary conditions (all possible external metabolites were available for uptake). For each intracellular metabolite, we identified the flux distribution that maximizes its production rate using the linear programming package CPLEX 9.0.0 (ILOG, Paris, France). If the maximal production rate of a given metabolite was zero, we considered it as a dead-end metabolite and not included in the set of producible metabolites.

Second, some biosynthetic pathways leading to producible metabolites involve reaction steps in which a non-producible cofactor participates (such a situation can occur if synthesis of the cofactor is incomplete in the reconstruction, but there is no net consumption of the cofactor by the pathway). As certain intermediates of these pathways would appear inaccessible in the scope analysis, we excluded them from the set of producible metabolites (even though they could be synthesized in steady state).

### Scope analysis

In the first step of scope analysis [17], metabolites produced in reactions whose substrates are all present in the initial seed are added to the initial seed to form the seed set for the next step. In successive steps, metabolites that can be produced from metabolites already present in the set are added to the seed set. The expansion of the seed set is finished when no new compounds can be added, that is, there are no reactions in the metabolic network whose substrate molecules are all in the seed set, but at least one of the products is not. The final set of molecules is referred to as the scope of the input set.

### Identifying autocatalytic compounds

If the scope of the input set did not include all metabolites that can be otherwise produced by the network, then we identified the smallest set of internal molecules that had to be added to the input set, so that the scope of this combined input included all required metabolites. To find the smallest set of such internal molecules, we searched for the metabolite that increased the scope to the highest extent (that is, a greedy algorithm). Next, we added this metabolite to the set of input molecules and performed the scope analysis again. The above steps were iterated until we arrived at an input set whose scope included all required metabolites.

Those molecules increasing the scope the most at various steps of the above procedure are either autocatalytic molecules (Figures S2, S3, S5 and S6 in Additional data file 1) or intermediates in the biosynthetic pathways leading to such molecules (Figure S5 in Additional data file 1). In other cases, the identity of the autocatalytic molecule is not self-evident from those compounds found to give the highest increase in the scope (Figures S1 and S7-S9 in Additional data file 1). In such cases, we analyzed the set of molecules, which became accessible after the addition of the identified molecule to the seed. These cases are further discussed in the description of the analysis of *Synechocystis *(Additional data file 1).

## Abbreviations

CoA, coenzyme A; THF, tetrahydrofolate.

## Authors' contributions

ESz conceived the idea for the study. All authors contributed to the design and planning of the research. BP performed the Flux Balance Analyses, and ÁK performed the scope analyses and network curations. All authors were involved in writing the manuscript. All authors approved the final version of the manuscript.

## Additional data files

The following additional data are available. Additional data file 1 includes details of the scope analysis for each organism and information on the reconstruction of the *Synechocystis *network. The figures (S1-S9) show the autocatalytic production of the identified metabolites. The table (S2) lists the relevant statistics for each metabolic network analyzed. Additional data file 2 is an Excel table presenting the list of producible metabolites for each metabolic network. If the network reconstruction used abbreviated names for the metabolites, then the abbreviations are also included for ease of comparisons with the reconstruction. Additional data file 3 is an Excel table describing the metabolic reconstruction of *Synechocystis *sp PCC 6803. The separate worksheets lists are: 1, the metabolites involved in the metabolic reconstruction, with their abbreviations and identifier used in the MetaCyc database; 2, the reactions with reaction ID, pathway, EC number and references; 3, the metabolites that cannot be produced in the network (dead end metabolites); 4, the reactions that were left out from the metabolic network, and the reason for the exclusion; and 5, the references and notes for the worksheets.

## Supplementary Material

Additional data file 1 The figures (S1-S9) show the autocatalytic production of the identified metabolites. The table (S2) lists the relevant statistics for each metabolic network analyzed. Click here for file

Additional data file 2 If the network reconstruction used abbreviated names for the metabolites, then the abbreviations are also included for ease of comparisons with the reconstruction. Click here for file

Additional data file 3 The separate worksheets lists are: 1, the metabolites involved in the metabolic reconstruction, with their abbreviations and identifier used in the MetaCyc database; 2, the reactions with reaction ID, pathway, EC number and references; 3, the metabolites that cannot be produced in the network (dead end metabolites); 4, the reactions that were left out from the metabolic network, and the reason for the exclusion; and 5, the references and notes for the worksheets. Click here for file
