# Supplementary material for: Computational identification of obligatorily autocatalytic replicators embedded in metabolic networks
Source: Genome Biol. 2008 Mar 10;9(3):R51. doi: 10.1186/gb-2008-9-3-r51 (PMC2397503; doi:10.1186/gb-2008-9-3-r51)
Supplement: Additional data file 1 — The figures (S1-S9) show the autocatalytic production of the identified metabolites. The table (S2) lists the relevant statistics for each metabolic network analyzed. [file gb-2008-9-3-r51-S1.doc]

Additional File 1, Table S1 and Figures S1-S9

**Computational identification of obligatorily autocatalytic replicators embedded in metabolic networks**

Ádám Kun^1,2^, Balázs Papp^3,4^ and Eörs Szathmáry^1,2,5^

*^1^ Collegium Budapest, Institute for Advanced Study, Szentháromság utca 2, Budapest H-1014, Hungary*

*^2^ Department of Plant Taxonomy and Ecology, Institute of Biology, Eötvös University, Pázmány Péter sétány 1/C, Budapest H-1117, Hungary*

*^3^ Faculty of Life Sciences, The University of Manchester, Michael Smith Building, Oxford Road, Manchester M13 9PT, United Kingdom*

*^4^ Present address: Institute of Biochemistry, Biological Research Center, Szeged H-6701, Hungary*

*^5^ Parmenides Center for the Study of Thinking, 14a Kardinal Faulhaber Strasse, D-80333 Munich, Germany*

### Brief description of the contents

We have analyzed the metabolic networks of eight eubacteria (*Escherichia coli, Heliobacter pylori, Staphylococcus aureus, Lactococcus lactis, Streptomyces coelicolor,* *Geobacter sulfurreducens, Mycobacterium tuberculosis* and *Synechocystis* sp.) an archaebacterium (*Methanosarcina barkeri*) and a eukaryote (*Saccharomyces cerevisiae*). The Additional Table S1 below lists the relevant statistics for each metabolic network analyzed. Next, the details of the scope analysis for each organism are presented. Information on the reconstruction procedure of the *Synechocystis* network is also described here.

**Additional Table S1. List of investigated metabolic networks and their main properties.**

| **Organism** | **Total number of reactions** | **Total number of metabolites** | **Number of producible metabolites (maximum scope)** | **Number of food molecules** | **Number of macromolecules** | **Scope of input metabolites** | **Additional metabolites to include for maximum scope^1^** |
| --- | --- | --- | --- | --- | --- | --- | --- |
| *Escherichia coli* | 932 | 761 | 692 | 143 | 3 | 315 | ATP |
| *Heliobacter pylori* | 478 | 485 | 441 | 74 | 5 | 182 | ATP |
| *Staphylococcus aureus* | 645 | 644 | 543 | 83 | 16 | 194 | ATP |
| *Lactococcus lactis* | 621 | 508 | 477 | 92 | 1 | 190 | ATP |
| *Streptomyces coelicolor* | 699 | 601 | 562 | 104 | 4 | 267 | ATP |
| *Mycobacterium tuberculosis* | 936 | 830 | 642 | 87 | 9 | 235 | ATP |
| *Saccharomyces cerevisiae* | 803 | 672 | 667 | 101 | 4 | 342 | ATP, ATP(m) |
| *Methanosarcina barkeri* | 619 | 628 | 566 | 70 | 21 | 161 | ATP + NAD^+^ |
| *Geobacter sulfurreducens* | 523 | 541 | 406 | 41 | 7 | 82 | ATP + NAD^+^ + THF + CoA |
| *Synechocystis*^2^ | 916 | 879 | 634 | 18 | 34 | 64 | ATP + NAD^+^ + THF + CoA + sugar |
| *Synechocystis*^3^ | 916 | 879 | 662 | 29 | 34 | 99 | ATP + NAD^+^ + THF + CoA |
| Minimal metabolism | 55 | 68 | 68 | 11 | 0 | 11 | ATP |

^1^ For a list of equivalent molecules that give the same scope see “Details of the scope analysis for each organism”

^2^ Autotrophic growth

^3^ Heterotrophic growth

# Details of the scope analysis for each organism

## 1. *Escherichia coli* K-12

*E. coli* has a well studied metabolism, thus the metabolic reconstruction available from Reed et al. [25] (http://gcrg.ucsd.edu/organisms/ecoli/ecoli_reactions.html) was used as is.

### Growth on rich medium

We added 3 macromolecules (acyl carrier protein, oxidized thioredoxin, Glu-tRNA) to the list of externally available molecules to define the initial seed for the scope analysis. The scope of this initial seed set of molecules does not contain all producible metabolites. However, if either ATP, ADP, ADP-glucose or 5-Phospho-alpha-D-ribose 1-diphosphate (prpp) is added to the seed, then all producible metabolites are within the scope. Importantly, ATP can be easily reached from those molecules (ADP, ADP-glucose and prpp) that are equivalent to ATP in their ability to extend the scope of the initial seed (apart from the above listed metabolites, the reactions by which ATP can be reached utilize only molecules within the scope of the initial seed):

2 ADP ↔ ATP + AMP (EC-2.7.4.3)

ADP-glucose → ADP + glycogen +H^+^ (EC-2.4.1.21)

AMP + H^+^ + prpp ↔ ATP + alpha-D-Ribose 5-phosphate (EC-2.7.6.1)

Thus, we conclude that ATP is a key autocatalytically produced metabolite.

### Growth on minimal medium

**Additional Figure S1. Autocatalytic synthesis of NAD^+^ in *Escherichia coli* (on minimal medium).** Metabolite increasing the scope so that it includes NAD^+^ are bold faced. Dashed line indicate the step in which NADH is used.

The set of externally available molecules were restricted to inorganic compounds and glucose. We added the same 3 macromolecules to the initial seed as above. First, we have to add ATP, ADP or ADP-glucose to increase the scope of the initial seed. However, only very few producible metabolites are within the scope of this seed. The seed has to be supplanted by other molecules, indicating that there are more autocatalytic cycles operating in the organism when grown on a minimal medium.

Second, we added (S)-2-[5-Amino-1-(5-phospho-D-ribosyl)-imidazole-4-carboxamido]-succinate (25aics) to the initial seed. While 25aics is an intermediate in the THF biosynthesis, in the reaction catalysed by the phosphoribosyl-aminoimidazole-succino-carboxamide synthase (E.C. 6.3.2.6) aspartate is released from 25aics, and in turn aspartate is the starting point for NAD^+^ biosynthesis. Thus, NAD^+^ is an autocatalytic molecule (Additional Fig. S1). The set of autocatalytic molecules equivalent to NAD^+^ (i.e. those that give the same scope as NAD^+^) also includes 4-phospho-L-aspartate, L-asparagine, L-aspartate, iminoaspartate, quinolinate, nicotinate-D-ribonucleotide, deamino-NAD^+^, nicotinamide, nicotinate, NMN, NADP^+^, NADPH, and NADH.

Third, we have to add CDPdiacylglycerol (E coli), which yields CTP in the reaction catalyzed by CDP-diacylglycerol synthetase (Ecoli) (EC-2.7.7.41). CTP is an intermediate in the biosynthesis of quinones, which are required cofactors in the *de novo* synthesis of pyrimidine nucleotides (Additional Fig. S2). Thus, quinones (menaquinone-8 or ubiquinone-8) are autocatalytic metabolites.

**Additional Figure S2. Autocatalytic synthesis of quinones in *Escherichia coli* (on minimal medium).** Metabolites increasing the scope so that it includes quinones are bold faced. Dashed line indicate the step in which quinones are used.

The set of autocatalytic molecules equivalent to quinone also include 2-demethylmenaquinone 8, 2-demethylmenaquinol 8, ubiquinol-8, ubiquinone-8, menaquinol 8, menaquinone 8, Orotate, orotidine 5'-phosphate, UMP, UDP, UTP, CTP, CDP, CMP, dCMP, dCDP, dCTP, dUMP, dUDP, dUTP, deoxyuridine, deoxycytidine, cytidine, cytosine, 2-phospho-4-(cytidine 5'-diphospho)-2-C-methyl-D-erythritol, 2-phospho-4-(cytidine 5'-diphospho)-2-C-methyl-D-erythritol, 2-C-methyl-D-erythritol 2,4-cyclodiphosphate, 1-hydroxy-2-methyl-2-(E)-butenyl 4-diphosphate, isopentenyl diphosphate, all-trans-octaprenyl diphosphate, CDPethanolamine, UDPgalactose, UDPglucose, uracil, uridine, UDP-N-acetyl-D-glucosamine, UDP-2,3-bis(3-hydroxytetradecanoyl)glucosamine, UDP-3-O-(3-hydroxytetradecanoyl)-D-glucosamine, UDP-3-O-(3-hydroxytetradecanoyl)-N-acetylglucosamine, 2-octaprenyl-6-hydroxyphenol, 2-octaprenyl-6-methoxy-1,4-benzoquinol, 2-octaprenyl-6-methoxyphenol, 2-octaprenylphenol, 2-octaprenyl-3-methyl-5-hydroxy-6-methoxy-1,4-benzoquinol, 2-octaprenyl3-methyl-6-methoxy- 1,4-benzoquinol, and 3-octaprenyl-4-hydroxybenzoate.

Fourth, and last we have to add one of the following molecules N-((R)-4-phosphopantothenoyl)-L-cysteine, acetyl-CoA, Coenzyme A, dephospho-CoA, malonyl-CoA, (R)-methylmalonyl-CoA, (S)-methylmalonyl-CoA, pantetheine 4'-phosphate, propanoyl-CoA, O-succinylbenzoyl-CoA, succinyl-CoA, which are either intermediates of the Coenzyme A biosynthesis route or Coenzyme A with an attached molecule. Thus, Coenzyme A can be considered as an autocatalytic molecule (see Additonal Fig. S4 below).

## 2. *Heliobacter pylori*

Metabolic reconstruction from Thiele et al. [45] (http://gcrg.ucsd.edu/organisms/hpylori.html) was used without modification. The list of externally available molecules is supplemented with 5 macromolecules (acyl carrier protein; oxidized thioredoxin; oxidized ferredoxin; Glu-tRNA and ferrocytochrome c-553) to define the initial seed for the scope analysis. The scope of these molecules does not contain all producible metabolites. However, all producible metabolites are within the scope of the seed if we add ATP, ADP or 5-Phospho-alpha-D-ribose 1-diphosphate (prpp) to the list of input molecules. Thus, we conclude that ATP production is autocatalytic (see also the results on *E. coli*).

## 3. *Staphylococcus aureus* N315

Metabolic reconstruction from Becker and Palsson [46] (http://gcrg.ucsd.edu/organisms/staph.html) was used without modification.

The list of externally available molecules is supplemented with 16 macromolecules (acyl carrier protein; oxidized thioredoxin; oxidized ferredoxin; lipoylprotein and 12 charged tRNAs) to define the initial seed for the scope analysis. The scope of these molecules does not contain all metabolites. However, all producible metabolites are within the scope of the seed if we add ATP, ADP, ADP-glucose, or 5-Phospho-alpha-D-ribose 1-diphosphate (prpp) to the list of input molecules. Thus, we conclude that ATP production is autocatalytic (see also the results on *E. coli*).

## 4. *Lactococcus lactis*

Metabolic reconstruction from Oliveira et al. [48] (http://www.biomedcentral.com/content/supplementary/1471-2180-5-39-S1.xls) was used with two minor modifications.

The following two reactions were changed to be irreversible:

ATP + H^+^ + nicotinate D-ribonucleotide → diphosphate + deamino-NAD^+^

ATP + H^+^ + pantetheine 4'-phosphate → diphosphate + dephospho-CoA

The reaction catalyzed by the nicotinic acid mononucleotide adenylytransferase (EC 2.7.7.18) is proved to be reversible in a biochemical assay in *E. coli* [53]. However, as the *E. coli* reconstruction considers this reaction as irreversible under physiological conditions, we assumed it is also irreversible in *L. lactis*. Similarly, the dephospho-CoA kinase (EC 2.7.1.24) was assumed to be irreversible under physiological conditions.

The list of externally available molecules is supplemented with oxidized thioredoxin to define the initial seed for the scope analysis. The scope of these molecules does not contain all producible metabolites. However, all producible metabolites are within the scope of the seed if we add ATP, ADP or 1-(5-Phosphoribosyl)-ATP to the list of input molecules. 1-(5-Phosphoribosyl)-ATP yields ATP in the reaction catalyzed by ATP phosphoribosyltransferase (EC 2.4.2.17):

1-(5-Phosphoribosyl)-ATP + diphosphate ↔ ATP + prpp

Thus, we conclude that ATP production is autocatalytic.

## 5. *Streptomyces coelicolor* A3(2)

Metabolic reconstruction from Borodina et al. [49] (http://www.genome.org/cgi/content/full/15/6/820/DC1) was used with minor modifications. As above, we considered argininosuccinate synthase (EC 6.3.4.5) to be irreversible as found in other manual reconstructions (e.g. *E. coli* [25], *H. pylori* [45], *L. lactis* [48]).

The list of externally available molecules is supplemented with oxidized thioredoxin, ferricytochrome c, acyl carrier protein and lipoylprotein to define the initial seed for the scope analysis. The scope of these molecules does not contain all producible metabolites.

However, all producible metabolites are within the scope of the seed if we add ATP, ADP, or 5-Phospho-alpha-D-ribose 1-diphosphate (prpp) to the list of input molecules. Thus, we conclude that ATP production is autocatalytic (see also the results on *E. coli*).

## 6. *Mycobacterium tuberculosis* H37Rv

Metabolic reconstruction from Jamshidi and Palsson [50] (http://gcrg.ucsd.edu/organisms/mtb/mtb.html) was used without modifications.

The list of externally available molecules is supplemented with oxidized ferrodoxin (2[4Fe-4S]), oxidized ferredoxin, oxidized adrenal ferredoxin, oxidized thioredoxin, ferricytochrome c, acyl carrier protein (apoprotein), tRNA^Ala^, tRNA^Glu^ and lipoylprotein to define the initial seed for the scope analysis. The scope of these molecules does not contain all producible metabolites.

However, all producible metabolites are within the scope of the seed if we add dGTP to the list of input molecules, which in turn yields ATP as follows:

dGTP + h2o → deoxyguanosine + inorganic triphosphate (EC-3.1.5.1)

AMP + inorganic triphosphate ↔ ADP + diphosphate (EC-2.7.4.3)

2 ADP ↔ ATP + AMP (EC-2.7.4.3)

Thus, we conclude that ATP production is autocatalytic.

## 7. *Saccharomyces cerevisiae*

Metabolic reconstruction iLL672 from Kuepfer et al. [47] (http://www.genome.org/cgi/content/full/15/10/1421/DC1) was used with one modification. The reaction catalyzed by the argininosuccinate synthase (EC-6.3.4.5) was considered to be irreversible [54,55]:

Asp + ATP + L-citrulline → AMP + N(omega)-(L-Arginino)succinate + H^+^ + ppi

The list of externally available molecules is supplanted by 4 macromolecules (cytoplasmic and mitochondrial oxidized thioredoxin; ferricytochrome c; and dolichol) and mitochondrial hydrogen ion to define the initial seed for the scope analysis. The scope of these molecules does not contain all producible metabolites.

Of all possible internal metabolites, the addition of mitochondrial ATP or mitochondrial ADP to the seed increased the scope the most. However, the scope of the expanded seed still does not contain all producible molecules (among others, it does not include cytoplasmic ATP).

The next and final metabolites to add are one of the followings: cytoplasmic ATP, cytoplasmic ADP and cytoplasmic or mitochondrial 5-Phospho-alpha-D-ribose 1-diphosphate.

Thus, we conclude that there is a separate autocatalytic cycle for cytoplasmic and mitochondrial ATP production.

## 8. *Geobacter sulfurreducens*

Metabolic reconstruction from Mahadevan et al. [52] (http://aem.asm.org/cgi/content/full/72/2/1558/DC1) was used with some modifications. THF biosynthesis was made complete with the reactions catalyzed by the GTP cyclohydrolase, dihydroneopterin triphosphate pyrophosphatase, dihydroneopterin monophosphate dephosphorylase and dihydroneopterin aldolase. The biosynthetic pathway was derived from the MetaCyc database [15,56].

The list of externally available molecules is supplemented with 7 macromolecules (acyl carrier protein; oxidized thioredoxin; ferrodoxin (oxidized form 4:2); ferricytochrome c; lipoylprotein and tRNAs charged with glutamine and glutamate) to define the initial seed for the scope analysis. The scope of these molecules does not contain all producible metabolites.

**Additional Figure S3. Autocatalytic synthesis of NAD^+^ in *Geobacter* *sulfurreducens*.** Metabolites increasing the scope so that it includes NAD+ are bold faced. Dashed line indicate the step in which NADH is used.

Of all possible internal metabolites, the addition of ADP-glucose to the seed increased the scope the most. However, the scope of the expanded seed still does not contain all producible molecules.

The next metabolites to add are any of the following metabolites: NAD^+^, NADH, deamino-NAD^+^, iminoaspartate, quinolinate and nicotinate D-ribonucleotide. In the metabolic reconstruction of *G. sulfurreducens* aspartate oxidase (NadB) was annotated as using NAD^+^ as a hydrogen acceptor, thus the biosynthesis of NAD^+^ is autocatalytic (Additional Fig. S3). In *E. coli* aspartate oxidase can use oxygen, FAD or fumarate as hydrogen acceptor [57]. However, being an anaerobic bacterium, *G. sulfurreducens* cannot utilize oxygen. Lovley and coworkers assumed that aspartate reductase utilizes NAD^+^ [52], as FAD is not found in this species (R. Mahadevan, personal communication). However, utilization of fumarate was not considered. If future experiments reveal that fumarate can be a hydrogen acceptor in the reaction converting L-aspartate to iminoaspartate, then NAD^+^ biosynthesis will no longer be regarded as autocatalytic in this species.

Please note that adding ATP and NAD^+^ to the initial seed results in the same scope as the addition of ADP-glucose and NAD^+^. However, this scope still does not cover all producible metabolites.


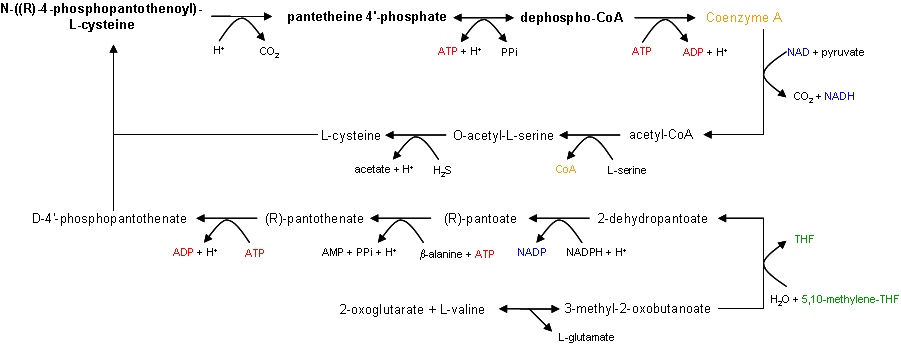


**Additional Figure S4. Autocatalytic synthesis of Coenzyme A in *Geobacter* *sulfurreducens****.* Metabolite increasing the scope so that it includes Coenzyme A are bold faced.

The next metabolite to add is N-((R)-4-Phosphopantothenoyl)-L-cysteine, which is an intermediate in the biosynthesis of Coenzyme A. N-((R)-4-Phosphopantothenoyl)-L-cysteine is formed from L-cysteine and D-4'-phosphopantothenate. The biosynthesis of L-cysteine requires Coenzyme A (Additional Fig. S4), consequently the biosynthesis of Coenzyme A is autocatalytic. The set of autocatalytic molecules equivalent to CoA also include panthetheine 4’-phosphate, dephospho-CoA, O-succinylbenzoyl-CoA, acetyl-CoA, isovaleryl-CoA, succinyl-CoA and malonyl-CoA. The scope of the initial seed complemented by ATP, NAD^+^ and CoA still does not contain all producible metabolites.

**Additional Figure S5. Autocatalytic synthesis of THF in *Geobacter* *sulfurreducens*.** Metabolites increasing the scope so that it includes THF are bold faced. Dashed line indicate the step in which THF is used.

In order to extend the scope to all producible metabolites, THF or intermediates in its biosynthesis (folate, GMP, GDP, GTP, IMP, dihydropteroate, 7,8-dihydrofolate, dihydro-neo-pterin, 2-amino-4-hydroxy-6-hydroxymethyl-7,8-dihydropteridine, 5-formamido-1-(5-phospho-D-ribosyl)-imidazole-4-carboxamide, dihyrdo-neo-pterin-phosphate, 5,10-methylenetetrahydrofolate, 5,10-methenyltetrahydrofolate, 5-formyltetrahydrofolate, 10-formyltetrahydrofolate) should be added to the initial seed.

There are two THF (10-formyl-THF) requiring steps in the *de novo* synthesis of GTP (Additional Fig. S5). Moreover, GTP is the starting molecule in the biosynthetic pathway leading to THF. Thus, THF is produced through an autocatalytic set of reactions.

In summary, the set of input molecules have to be supplemented with ATP and the coenzymes NAD^+^, Coenzyme A and THF in order to reach all producible metabolites.

## 9. *Methanosarcina barkeri*

Metabolic reconstruction from Feist et al. [51] (http://gcrg.ucsd.edu/organisms/mbarkeri.html) was used without modification.

The list of externally available molecules is supplemented with 21 macromolecules (oxidized thioredoxin; oxidized ferredoxin and 19 charged tRNAs) to define the initial seed for the scope analysis. The scope of these molecules does not contain all producible metabolites.

Of all possible internal metabolites, the addition of ATP or ADP to the seed increased the scope the most. However, the scope of the expanded seed still does not contain all producible molecules.

The highest increase in the scope can be obtained by the addition of any one of a large number of metabolites (NAD(H); NADP(H); D-Fructose 1-phosphate; D-Fructose 6-phosphate; D-glucose; D-glucose 1-phosphate; D-glucose 6-phosphate; glycogen; GDP-D-mannose; deamino-NAD^+^; nicotinate D-ribonucleotide; orotidine 5'-phosphate; D-fructose 1,6-bisphosphate; D-mannose 1-phosphate; mannose 6-phosphate; N-(5-phospho-D-ribosyl)anthranilate; 1-(2-carboxyphenylamino)-1-deoxy-D-ribulose 5-phosphate; D-ribulose 5-phosphate; D-xylulose 5-phosphate; dihydroxyacetone phosphate; C'-(3-Indolyl)-glycerol 3-phosphate; glyceraldehyde 3-phosphate; UDP-galactose; UDP-glucose; 5-phospho-alpha-D-ribose 1-diphosphate; and alpha-D-ribose 5-phosphate).

The key component is NAD^+^, which can be reached from all of the above molecules (Additional Fig. S6). NAD^+^ can be synthesized from 5-Phospho-alpha-D-ribose 1-diphosphate (prpp) and externally available nicotinic acid. Prpp is synthesized from ATP and alpha-D-ribose 5-phosphate. *M. barkeri* does not take up sugars of 5 and 6 carbon atoms. Its main carbon sources are methanol, acetate and pyruvate. In order to produce sugars of the pentose phosphate pathway, a reaction requiring NADH is necessary to convert 3-phospho-D-glyceroyl-phosphate to glyceraldehyde 3-phosphate (Additional Fig. S6).

**Additional Figure S6. Autocatalytic synthesis of NAD^+^ in *M. bakeri*.** Metabolites completing the scope are bold faced. Dashed line indicate the step in which NADH is used.

Thus, in addition to ATP, the biosynthesis of NAD^+^ is also autocatalytic in this species.

## 10. Synechocystis sp. strain PCC 6803

### Metabolic reconstruction

The complete genome of *Synechocystis* sp. strain PCC6803 was sequenced in 1996 [58]. Our metabolic reconstruction was based on an automated reconstruction generated from the annotated genome and deposited in the MetaCyc database [15,56]. In addition to this automated reconstruction, a manually curated reconstruction was also available for the central metabolism of *Synechocystis* sp. strain PCC6803 [59].

Experiments demonstrate that cyanobacteria can grow under autotrophic conditions (i.e. on mineral nutrients, CO_2_ and light). This observation also implies that *Synechocystis* sp. strain PCC6803 can synthesize all of its components from these food molecules. The automatically annotated set of reaction was manually curated for reaction reversibility, stochiometric completeness and cofactor usage. Gaps in the metabolic network were filled (see below). We have also incorporated reactions from the literature. In total, we have added 139 reactions to the automated metabolic reconstruction.

**Supplemental Figure S7. Autocatalytic metabolites in *Synechocystis* sp.: ATP and D-ribulose-1,5-biphosphate.** Reaction paths leading from ADP-ribose and ADP-glucose to ATP and the Calvin-cycle.

The biosynthesis of all amino acids (except for lysine) is reported to be complete for this genome in the KEGG database [60]. We added two reactions to fill gaps in the asparagine and cysteine biosynthesis. Furthermore, while tyrosine biosynthesis was complete an alternative pathway leading from prephenate through L-arogenate to tyrosine also exists [61]. Active transport of nearly all amino acids were demonstrated [62], but its main function is to prevent loss due to leaking.

5 enzymes of Coenzyme-A biosynthesis are not yet identified in the genome, thus the missing reactions were added to complete the pathway (based on the *E. coli* metabolic network reconstruction).

Reactions for plastoquinone and vitamin-E biosynthesis [63] were added to the network (starting from p-hydroxi-phenylpyruvate).

One reaction was deleted from the automatically annotated reaction list. Two genes were annotated as glutathion peroxidases based on sequence similarity, but no gluthation peroxidase activity could be found in *Synechocystis* [64]. The two glutathione peroxidase like genes (Gpx-1 and Gpx-2) exhibit peroxidase activity, but utilize NADPH as electron donor [65].

### Autotrophic growth

The list of externally available molecules is supplemented with 34 molecules not synthesized by the network (acyl carrier protein; oxidized ferredoxin; oxidized rubredoxin; oxidized thioredoxin; ferricytochrome-B1; ferrocytochrome-c; pimelate; dolichol phosphate; polyphosphate; oxidized flavodoxin; 1-4-alpha-D-Glucan; THF-glutamine polymer; formyl-THF-glutamine polymer and 21 tRNAs) to define the initial seed for the scope analysis. The scope of these molecules does not contain all producible metabolites.

**Additional Figure S8. Autocatalytic synthesis of NAD^+^ in *Synechocystis* sp. PCC 6803.** Metabolites increasing the scope so that it includes NAD+ are bold faced. Dashed line indicate the step in which NAD+/NADH is used.

First, we have to add ADP-ribose or ADP-glucose to the initial seed. This molecule can lead to ATP (ADP) and sugars, two autocatalytic sets of molecules (Additional Fig. S7).


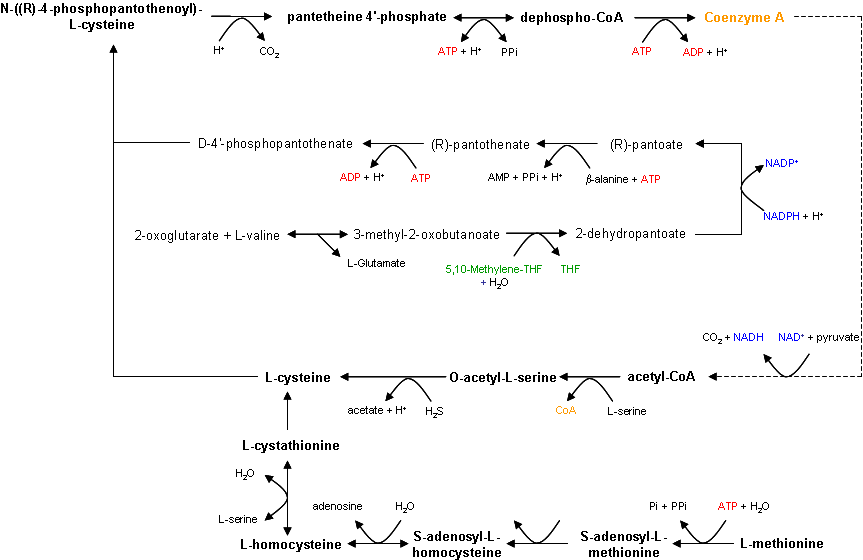


**Additional Figure S9. Autocatalytic synthesis of Coenzyme A in *Synechocystis* sp. PCC 6803.** Metabolites increasing the scope so that it includes Coenzyme A are bold faced. Dashed line indicate the step in which Coenzyme A is used.

Second, we have to add L-glutamine, L-glutamate or D-glutamate. Aspartate, a starting point for NAD^+^ biosynthesis, is formed from L-glutamate and oxalacetate, both of which require NAD^+^ for their production (Additional Fig. S8). L-glutamine participates in numerous trans-amination reactions as a cofactor, thus its scope is larger than the scope of NAD^+^, which does not include L-glutamine. 2-oxoglutarate, the precursor for L-glutamine, can only be formed if Coenzyme-A is also present. The set of autocatalytic molecules equivalent to NAD^+^/NADH includes L-glutamate, D-glutamate, L-glutamine, L-aspartate, L-asparagine, iminoaspartate, 5-amino-1-(5-phospho-D-ribosyl)imidazole-4-carboxamide, (S)-2-[5-amino-1-(5-phospho-D-ribosyl)imidazole-4-carboxamido]succinate, 4-phospho-L-aspartate, quinolinate, nicotinate D-ribonucleotide, deamino-NAD^+^, 4-(cytidine 5'-diphospho)-2-C-methyl-D-erythritol, and 2-phospho-4-(cytidine 5'-diphospho)-2-C-methyl-D-erythritol.

Finally, we have to add S-adenosyl-L-methionine, L-methionine or S-adenosyl-L-homocysteine. All these molecules can be converted to L-cysteine, an intermediate in the biosynthesis of Coenzyme A (Additional Fig. S9). The set of autocatalytic molecules equivalent to CoA also include N-((R)-4-phosphopantothenoyl)-L-cysteine, pantetheine 4'-phosphate, dephospho-CoA, O-succinylbenzoyl-CoA, acetyl-CoA, propanoyl-CoA, succinyl-CoA, pimeloyl-CoA and malonyl-CoA.

Furthermore, during the conversion of the finally added metabolites S-adenosyl-L-homocysteine to L-homocysteine (Additional Fig. S9), adenosine is released, which can be converted to GTP, the starting molecule in THF biosynthesis (Additional Fig. S5). However, we do not consider methionine as an autocatalytic molecule: in the normal biosynthetic pathway it is produced from L-homocysteine with THF as cofactor. The set of autocatalytic molecules equivalent to THF includes 5-methyltetrahydrofolate, 5-formyltetrahydrofolate, 5,10-methenyltetrahydrofolate, 5,10-methylenetetrahydrofolate, 10-formyltetrahydrofolate, GTP, GDP, GMP, XMP, IMP, L-methionine, guanine, guanosine, adenine, adenosine, S-adenosyl-L-methionine, S-adenosyl-L-homocysteine, 5-formamido-1-(5-phospho-D-ribosyl)imidazole-4-carboxamide, 7,8-dihydrofolate, inosine, hypoxanthine, GDP-4-dehydro-6-deoxy-D-mannose, dihyrdo-neo-pterin-phosphate, dihydro-neo-pterin, dihydropteroate, dihydroneopterin monophosphate, 6-hydroxymethyl-dihydropterin pyrophosphate, 6-hydroxymethyl dihydropterin and GDP-L-galactose.

While we have to add only 3 metabolites to complete the scope, there are more autocatalytic cycles than suggested by these 3 molecules. The addition of ATP, NAD^+^, Coenzyme A, THF and glyceraldehyde 3-phosphate (for example) to the initial seed results in the same scope as the addition of the 3 molecules identified in the scope analysis (see above). If the initial seed contains ATP, NAD^+^, Coenzyme A and THF then the required sugar component can be any one of 138 molecules, including components of the pentose phosphate pathway, nucleotides, amino acids, etc. (the full list is as follows: 3-phospho-D-glyceroyl phosphate, (S)-2-[5-amino-1-(5-phospho-D-ribosyl)imidazole-4-carboxamido]succinate, (S)-2-aceto-2-hydroxybutanoate, 1-(2-carboxyphenylamino)-1-deoxy-D-ribulose 5-phosphate , 2-deoxy-D-ribose 1-phosphate , 2-deoxy-D-ribose 5-phosphate, 2-phospho-4-(cytidine 5'-diphospho)-2-C-methyl-D-erythritol, D-glycerate 2-phosphate, C'-(3-indolyl)-glycerol 3-phosphate, 3-phospho-D-glycerate, 5-O-(1-carboxyvinyl)-3-phosphoshikimate, 3-sulfinyl-pyruvate , 4-amino-4-deoxychorismate, 4-(cytidine 5'-diphospho)-2-C-methyl-D-erythritol, 4-phospho-L-aspartate, 6-phospho-D-gluconate, 8-Amino-7-oxononanoate, acetyl-ACP, aAcetaldehyde, acetate, acetyl-CoA, N-acetyl-D-glucosamine 1-phosphate, N-acetyl-D-glucosamine 6-phosphate, N-acetyl-L-glutamate, O-acetyl-L-serine, acetylphosphate , acyl-SN-glycerol-3-phosphate, adenosine, ADPglucose, ADP-ribose, S-adenosyl-L-homocysteine, D-alanyl-D-alanine, α-D-glucose, D-arabinose 5-phosphate, N(omega)-(L-Arginino)succinate, L-Aspartate 4-semialdehyde, chorismate, L-cystathionine, Cytidine, D-glucono-d-lactone-6-phosphate , D-arabino-3-hexulose 6-P , CTP, CDP, CMP, dCDP, dCMPcm, dCTP, dTDP, dTMP, dTTP, dUDP, dUMP, dUTP, GMP, GDP, GTP, UMP, UDP, UTP, Deoxycytidine, dihydroxyacetone, dihydroxyacetone phosphate, dipicolinate, Deoxyuridine, ethanol, D-fructose 6-phosphate, 5-formamido-1-(5-phospho-D-ribosyl)imidazole-4-carboxamide, D-fructose, fumarate, D-glucose 1-phosphate, Glyceraldehyde 3-phosphate, D-glucose 6-phosphate, D-glucose 6-phosphate, GDP-L-galactose, GDP-D-mannose, D-glucose, glycerol, 2-(β-D-glucosyl)-sn-glycerol-3-phosphate, Glycogens, (R)-glycerate, guanosine, L-homoserine, Hydroxypyruvate, Inosine-5’-phosphate, Inosine, sn-Glycero-3-phosphoethanolamine, L-1-phosphatidyl-glycerol , L-1-phosphatidylglycerol-phosphate, L-1-phosphatidil-serine, D-lactate, L-asparagine, L-aspartate, L-alanine, L-threonine, L-threonine, D-alanine, (R)-S-lactoylglutathione, maltose, malonyl-[acyl-carrier protein], malonyl-CoA, maltodextrin, maltotetraose, D-mannose, D-mannose 1-phosphate, D-mannose 6-phosphate, oxaloacetate, orotidine 5'-phosphate, Phosphoenolpyruvate, O-phospho-L-homoserine, N-(5-phospho-D-ribosyl)anthranilate, 5-phospho-alpha-D-ribose 1-diphosphate, pyruvate, alpha-D-ribose 1-phosphate, alpha-D-Ribose 5-phosphate, D-Ribulose 5-phosphate, D-sorbitol 6-phosphate, sucrose 6-phosphate, sucrose, thymidine, UDP-3-O-(3-hydroxytetradecanoyl)-N-acetylglucosamine, UDP-N-acetyl-D-glucosamine, UDPglucose, UDP-D-glucuronate, UDP-N-acetyl-D-galactosamine, UDPgalactose, uridine, xanthosine 5'-phosphate, D-xylulose 5-phosphate, N6-(1,2-dicarboxyethyl)-AMP, D-fructose 1,6-bisphosphate, 2,3-Dihydrodipicolinate, UDP-2,3-bis(3-hydroxytetradecanoyl)glucosamine, 1-4-alpha-D-glucan, 6-alpha-D-1-4-alpha-D-glucano-glucan, D-ribulose-1,5-bisphosphate, N-Succinyl-LL-2,6-diaminoheptanedioate, and D-sedoheptulose-1,7-bisphosphate).

### Heterotrophic growth

We also considered heterotrophic growth for *Synechocystis* when uptake of certain organic molecules is possible. Even thought there are transport-reactions for the uptake of amino acids, physiologically these reactions are for the prevention of amino acid leakage [62], and thus amino acids were not added to the external food set. The expanded list of externally available molecules was supplemented with the same 34 macromolecules as above. The scope of these molecules does not contain all metabolites.

First, we have to add ADP-ribose, ADP-glucose, ADP or ATP to the initial seed (Additional Fig. S7).

Second, we have to add L-glutamine, L-glutamate or D-glutamate (Additional Fig. S8).

Finally, we have to add S-adenosyl-L-methionine, L-methionine or S-adenosyl-L-homocysteine (Additional Fig. S9).

In summary, heterotrophic growth yields the same set of autocatalytic molecules, with the notable exception of the Calvin-cycle.

## 11. Minimal metabolism

A hypothetical minimal metabolism suggested by Moya and coworkers [16] takes up D-glucose as a carbon source; guanine, adenine and uracil for the nucleotide bases and serine and palmitoyl-CoA for the biosynthesis of phospholipids. The suggested minimal reaction network was based on the metabolism of *Buchnera aphidicola*. In this organism the phosphorylation of the primary carbon source (glucose) requires phosphoenolpyruvate. We replaced this reaction with the more common reaction requiring ATP:

ATP + D-glucose → ADP + H^+^ + D-glucose-6-phosphate

The scope of the external metabolites does not contain all producible metabolites. ATP has to be added to the initial seed to make them accessible, therefore we conclude that ATP production is autocatalytic in this hypothetical minimal metabolism.
